# Orthostatic hypotension in hereditary transthyretin amyloidosis: epidemiology, diagnosis and management

**DOI:** 10.1007/s10286-019-00623-x

**Published:** 2019-08-26

**Authors:** Jose-Alberto Palma, Alejandra Gonzalez-Duarte, Horacio Kaufmann

**Affiliations:** 1 Department of Neurology, New York University School of Medicine, 530 First Avenue, Suite 9Q, New York, NY 10016 USA; 2 Departament of Neurology, Instituto Nacional de Ciencias Médicas y Nutrición Salvador Zubirán, Mexico D.F., Mexico

**Keywords:** Autonomic dysfunction, Autonomic failure, Orthostatic hypotension, Amyloid, Transthyretin, Peripheral neuropathy, Droxidopa

## Abstract

**Purpose:**

Neurogenic orthostatic hypotension is a prominent and disabling manifestation of autonomic dysfunction in patients with hereditary transthyretin (TTR) amyloidosis affecting an estimated 40–60% of patients, and reducing their quality of life. We reviewed the epidemiology and pathophysiology of neurogenic orthostatic hypotension in patients with hereditary TTR amyloidosis, summarize non-pharmacologic and pharmacological treatment strategies and discuss the impact of novel disease-modifying treatments such as transthyretin stabilizers (diflunisal, tafamidis) and RNA interference agents (patisiran, inotersen).

**Methods:**

Literature review.

**Results:**

Orthostatic hypotension in patients with hereditary transthyretin amyloidosis can be a consequence of heart failure due to amyloid cardiomyopathy or volume depletion due to diarrhea or drug effects. When none of these circumstances are apparent, orthostatic hypotension is usually neurogenic, i.e., caused by impaired norepinephrine release from sympathetic postganglionic neurons, because of neuronal amyloid fibril deposition.

**Conclusions:**

When recognized, neurogenic orthostatic hypotension can be treated. Discontinuation of potentially aggravating medications, patient education and non-pharmacologic approaches should be applied first. Droxidopa (Northera^®^), a synthetic norepinephrine precursor, has shown efficacy in controlled trials of neurogenic orthostatic hypotension in patients with hereditary TTR amyloidosis and is now approved in the US and Asia. Although they may be useful to ameliorate autonomic dysfunction in hereditary TTR amyloidosis, the impact of disease-modifying treatments on neurogenic orthostatic hypotension is still uninvestigated.

## Case vignette

A 44-year-old French-Canadian male presented with a 3-year history of severe diarrhea, unintentional weight loss of 10 kg in the last year and frequent episodes of lightheadedness and feeling about to faint when standing up. Upon further questioning he reported numbness and tingling with occasional shooting pains in his feet at night. He had surgery for left carpal tunnel syndrome at age 35 years. His father had similar symptoms but died at age 52 of lung cancer. Neurologic examination showed normal muscle strength, reduced pin and light touch sensation up to the ankles, very reduced in the calves and reduced in his fingers, bilaterally. Deep tendon reflexes were absent (Achilles and patellar) or reduced (bicipitalis, tricipitalis, brachioradialis). His blood pressure (BP) in the supine position was 139/61 mmHg with a heart rate of 71 bpm. After 3-min of standing, his BP decreased to 89/42 with a heart rate of 74 bpm, and he reported feeling very lightheaded. Plasma norepinephrine levels in the supine position were low (72 pg/ml) and failed to increase significantly on standing (89 pg/ml). Nerve conduction studies were consistent with a moderate axonal sensory neuropathy with no motor involvement. Genetic testing disclosed a heterozygous Val71Ala mutation in the *TTR* gene, and a colonic biopsy was positive for amyloid. Treatment with droxidopa (Northera^®^) 200 mg three times/day resulted in improvement in orthostatic tolerance and increased BP when standing. His diarrhea improved with diphenoxylate and atropine and tincture of opium.

## Introduction

Hereditary transthyretin amyloidosis is a rare, autosomal dominant disease caused by mutations in the *TTR* gene encoding transthyretin, a T4-thyroid hormone and retinol transport protein [107]. The liver is the primary source of circulating wild-type tetrameric transthyretin protein. The disease was first reported in Portugal in the 1950s [8] and is estimated to affect ~ 50,000 patients worldwide [107].

More than 150 *TTR* pathogenic mutations have now been identified, with the Val20Met mutation being the most frequent (~ 50% of cases). Mutations in the *TTR* gene destabilize the tetramer structure of transthyretin, turning it into monomers and fibrils that deposit as amyloid predominantly in the peripheral motor, sensory and autonomic nerves, gastrointestinal tract and heart resulting in progressive polyneuropathy and cardiomyopathy [107]. The disease is progressive, with survival of 2–15 years after the onset of neuropathy, but only 2–5 years in patients presenting with cardiomyopathy [23, 74, 86].

Autonomic dysfunction is prominent and disabling in patients with hereditary transthyretin amyloidosis and can be the presenting feature of the disease in ~ 10% of cases before the development of sensory-motor neuropathy or cardiomyopathy [2, 21, 37, 45, 101]. Among the most incapacitating features of autonomic dysfunction is orthostatic hypotension (OH), which is a sustained fall in blood pressure (BP) on standing. The current definition of OH, based on expert consensus [42], is a fall of at least 20 mmHg in systolic BP or 10 mmHg in diastolic BP within 3 min of standing or upright tilt. OH can impair perfusion to organs above the heart, most notably the brain, resulting in symptoms of tissue hypoperfusion, such as dizziness, lightheadedness, feeling about to faint and, sometimes, syncope. These symptoms are disabling, have a profound impact on a patient’s quality of life and are associated with worse survival.

This article reviews the current knowledge on the epidemiology, physiopathology and management of OH with emphasis on patients with hereditary transthyretin amyloidosis. We summarize non-pharmacologic and pharmacologic treatment strategies and discuss the impact of novel disease-modifying treatments such as transthyretin stabilizers (diflunisal, tafamidis) and RNA interference agents (patisiran, inotersen) on OH.

## Epidemiology of OH in hereditary TTR amyloidosis

Although the first patients reported in 1952 by Corino de Andrade [8] did have gastrointestinal abnormalities and genitourinary disturbances, OH was not specifically mentioned. To our knowledge, the first description of OH in patients with hereditary amyloidosis polyneuropathy was published in 1968 by Araki and colleagues in Japan [9].

The estimated prevalence of OH in patients with hereditary transthyretin amyloidosis is 40–60% [46, 80, 108]. It is frequent, early and severe in patients with the Val30Met mutation and early-onset disease but appears to be less severe in Val30Met cases with late-onset disease [70–72, 74, 108, 138]. OH is also prevalent and severe in patients with some non-Val30Met mutations [12, 15, 20, 24, 25, 27, 49, 53, 57, 60, 69, 73, 78, 79, 95, 96, 113, 115, 119, 136, 137, 142, 145]. For instance, up to 100% of patients with the Ala97Ser mutation have OH, with 71% having frequent syncope, particularly in late stages of the disease [58].

Conversely, OH appears to be infrequent in patients with *TTR* mutations with high prevalence in Scandinavian countries (e.g., Ala45Ser, Tyr69His, Leu111Met) [127], and in patients with the Val122Ile mutation, the most common *TTR* mutation in African Americans [114].

In a recent study involving > 3000 subjects enrolled in the multinational, longitudinal, observational Transthyretin Amyloidosis Outcomes Survey (THAOS), 58.7% had symptomatic OH [46]. Moreover, the severity of the fall in BP when standing appeared to worsen at annual follow-ups, reflecting the progressive nature of autonomic failure. More pronounced orthostatic BP reductions were associated with increasing age, worse polyneuropathy disability (mPND) stage and diarrhea [46].

## Pathophysiology of OH in hereditary TTR amyloidosis

OH in patients with hereditary transthyretin amyloidosis who do not have heart failure, volume depletion (frequently caused by diarrhea) or drug effects is usually neurogenic, i.e., caused by impaired norepinephrine release from sympathetic postganglionic neurons, because of neuronal amyloid fibril deposition. This has been documented by neuropathology, neuroimaging and measurements of the plasma concentration of catecholamines.

Autopsy studies in patients with hereditary transthyretin amyloidosis and severe OH showed amyloid-related degeneration of the peripheral autonomic nervous system, namely, anterior and posterior roots of the spinal cord, sympathetic ganglia, postganglionic sympathetic nerves and the vagus nerve. Neuronal density in the intermediolateral column of the spinal cord was reduced, and there was degeneration of sympathetic postganglionic cholinergic fibers. The brain was consistently unaffected [10, 24, 33, 83, 96, 101, 113, 119, 132, 143].

Studies with ^123^I-metaiodobenzyiguanidine (MIBG) cardiac neuroimaging showed reduced cardiac sympathetic innervation [34, 56, 97, 134], which can be present before any abnormal echocardiographic sign. Moreover, cardiac sympathetic denervation predicts worse prognosis [3, 31, 55, 120].

Plasma levels of norepinephrine, the main sympathetic neurotransmitter, are severely reduced and fail to increase when standing in patients with hereditary transthyretin amyloidosis. Moreover, administration of norepinephrine elicits noteworthy increases in heart rate and blood pressure, indicating sympathetic denervation supersenstivity [43, 128, 137, 144].

The mechanisms of neurogenic OH in hereditary transthyretin amyloidosis are similar to those of peripheral neurodegenerative synucleinopathies, i.e., Parkinson disease, dementia with Lewy bodies and pure autonomic failure, in which dysfunction of the sympathetic nerves is mediated by accumulation of another misfolded protein, α-synuclein, highlighting the high affinity that both misfolded transthyretin and α-synuclein have for the autonomic nervous system [104].

## Approach to the patient with hereditary TTR amyloidosis and neurogenic OH

Neurogenic OH can be symptomatic or asymptomatic. Typical symptoms of neurogenic OH are lightheadedness, dizziness, blurry vision and, when the fall in BP is pronounced, loss of consciousness and postural tone (syncope). Symptoms occur predominantly when standing, less frequently when sitting and always abate when lying down. Patients with nOH may also complain of generalized weakness, fatigue, leg buckling, occipital headache, neck and shoulder (“coat hanger”) discomfort, and shortness of breath due to ventilation/perfusion mismatch in the apical lung areas.

Patients with chronic nOH due to neurologic disorders usually tolerate very low BPs with only mild or no symptoms at all but syncope can occur with added orthostatic stressors (e.g., large carbohydrate-rich meals, alcohol intake, very warm weather, dehydration and antihypertensive treatment).

The morning hours tend to be most difficult as symptoms of nOH are aggravated by intravascular volume loss overnight [11]. Meals, particularly carbohydrate-rich, lead to splanchnic vasodilatation and post-prandial hypotension (i.e., fall in blood pressure within 2 h of eating) [118]. Physical inactivity and prolonged bed rest are common in patients with nOH. This leads to cardiovascular deconditioning further worsening the fall in BP and increasing symptoms leading to a vicious cycle.

In addition to nOH, other manifestations of autonomic failure in hereditary transthyretin amyloidosis include erectile dysfunction (ED), particularly in patients with early-onset of the disease (i.e., < 50 years old). The treatment of ED is challenging, as the use of sildenafil and other phosphodiesterase inhibitors may unmask or aggravate nOH. A good strategy is to screen for OH in all male patients with hereditary transthyretin amyloidosis before recommending sildenafil. Ideally, sildenafil should be administered at the office to determine its actual impact on blood pressure. For patients with severe nOH and ED, alternative treatments such as vacuum pumps and intracavernosal and intraurethral devices with or without prostaglandin E1 (PGE1) may be considered instead.

Moderate-severe cardiomyopathy can affect some patients with hereditary transthyretin amyloidosis. Cardiac failure in patients with OH can prevent the heart from pumping efficiently or rapidly enough to compensate for the fall in BP when standing, aggravating the orthostatic fall and potentially resulting in syncope. In these circumstances, treatment of heart failure with diuretics can further aggravate OH. Angiotensin-converting enzyme inhibitors, angiotensin receptor blockers and beta-blockers have not been well studied in amyloidosis, although they may exacerbate OH. If reducing the preload is imperative, low doses at night are preferable [93].

## Diagnosis of neurogenic OH

**Fig. 1:**
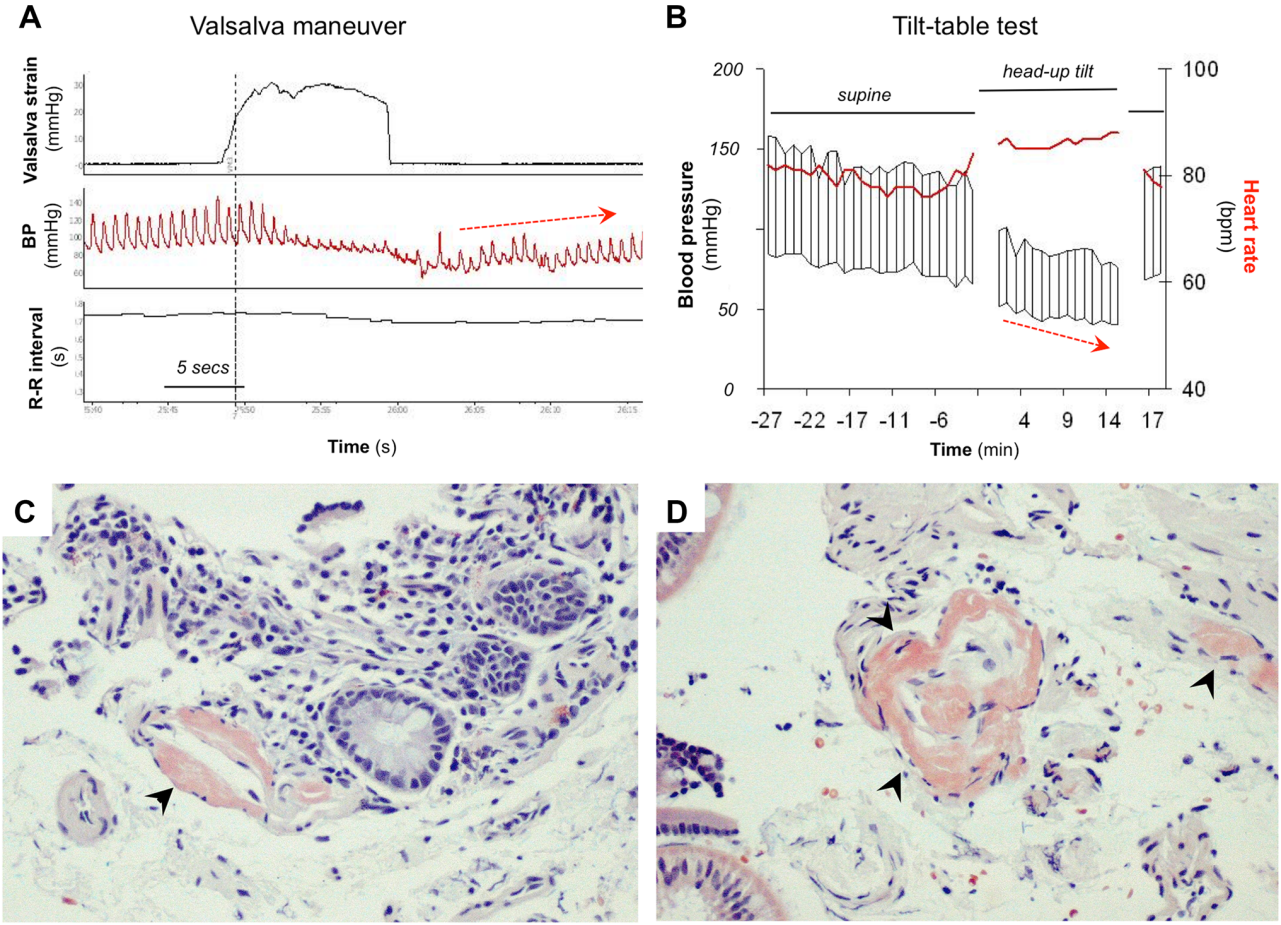
Cardiovascular autonomic testing and gastrointestinal biopsy in a patient with acquired amyloid polyneuropathy after domino liver transplant. **a**–**d** Representative test results of a 76-year-old female who, as a result of autoimmune liver failure, had a liver transplant at age 55 years. She suffered a liver transplant rejection and required a second liver transplant at age 69. The liver she received was from a donor with hereditary transthyretin amyloidosis (Val71Ala mutation), who had recently died after several years of severe sensory and autonomic neuropathy (i.e., domino liver transplant). Approximately 1 year after receiving the second liver transplant, the patient developed severe diarrhea, exercise intolerance and painful tingling in the feet. Two years after the liver transplant her tingling had worsened significantly and she developed dry mouth and neurogenic bladder. At age 75 she developed severe weight loss (15–20 lb), recurrent orthostatic dizziness and lightheadedness and suffered frequent episodes of loss of consciousness upon standing. She became wheelchair bound at age 76. **a** Cardiovascular autonomic testing showing absent blood pressure overshoot after release of the Valsalva strain (dashed arrow), indicating impaired baroreflex-mediated sympathetic activation. **b** Tilt-table test showing a supine blood pressure of 133/70 mmHg with a heart rate of 79 bpm. After 15 min of head-up tilt, her blood pressure had fallen to 76/40 mmHg and her heart rate was 88 bpm, consistent with severe neurogenic orthostatic hypotension. To confirm that her sensory and autonomic neuropathy was caused by amyloid, she underwent a upper gastrointestinal endoscopy and biopsies from the stomach and duodenum were obtained. **c** and **d** Congo red stain in upper gastrointestinal tissue showing abundant amyloid deposition in the muscularis mucosae (arrows)

The diagnosis of OH requires BP readings while supine and upright, either during active standing or during a tilt-table test, to determine the presence of a sustained orthostatic fall of at least 20 mmHg systolic or 10 mmHg diastolic BP. BP and heart rate should be measured after the patient has been supine for at least 5 min and after standing still (or passively tilted) for 1–3 min (Fig. 1).

The changes in heart rate on standing help to determine whether the OH is neurogenic in origin. In patients with nOH, reduced sympathetic innervation causes the heart rate to increase much less than expected considering the magnitude of the BP fall [41, 102]. Therefore, a blunted heart rate increase during hypotension suggests a neurogenic cause. A ratio between the increase in heart rate and fall in systolic BP upon standing or head-up tilt (**Δ**HR/**Δ**SBP ratio) < 0.5 bpm/mmHg is diagnostic of nOH [100]. Conversely, a **Δ**HR/**Δ**SBP ratio ≥ 0.5 suggests a non-neurogenic cause.

Ascertaining the diagnosis of nOH may require autonomic testing including the BP response to the Valsalva maneuver and plasma norepinephrine levels while supine and standing [6, 72, 73]. During the Valsalva maneuver, patients with nOH fail to show the classical BP “overshoot” after release of the strain (phase IV) (Fig. 1). An increase in plasma norepinephrine after 5–10 min of standing below 100% suggests defective baroreflex-mediated sympathetic activation and a diagnosis of nOH.

Ambulatory BP monitoring (ABPM) can help in the diagnosis and management of nOH [99]. Affected patients typically have a reversal of the normal circadian blood pressure pattern with higher BP during the night when the patient is supine in bed than during the day (i.e., non-dipping BP) [5, 22]. Nocturnal supine hypertension causes pressure natriuresis with exaggerated sodium and water loss causing overnight depletion of intravascular volume, worsening OH in the morning. ABPM and a detailed diary of activities are also useful to specifically tailor the use of short-acting pressor agents only at times when OH is severe in patients that may remain seated for long periods of the day or are wheelchair-bound.

## Management of neurogenic OH in hereditary TTR amyloidosis

The goal of treatment of nOH in patients with hereditary transthyretin amyloidosis is not to normalize standing BP, but to reduce symptom burden, improve quality of life and reduce the morbidity and mortality associated with nOH. Cardiomyopathy and heart failure are present in many patients. This can complicate the management of nOH, as treatment of heart failure typically involves reducing the cardiac preload with diuretics causing intravascular volume depletion and worsening nOH. Similarly, diarrhea, a manifestation of gastrointestinal involvement in hereditary tranthyretin amyloidosis, causes volume depletion, which aggravates nOH.

Consensus guidelines for the treatment of nOH are available [44, 77]. The steps of nOH management include: (1) correcting aggravating factors, (2) implementing non-pharmacologic measures and (3) drug therapies. When nOH is asymptomatic, treatment may not be required or may be limited to non-pharmacologic measures. When nOH is symptomatic, pharmacologic treatment is usually required.

### Correction of aggravating factors

Drugs that reduce intravascular volume (diuretics), induce vasodilatation (sildenafil, nitrates) or block norepinephrine release/activity at the neurovascular junction (α-blockers, centrally acting α_2_-agonists, tricyclic antidepressants) worsen nOH and symptoms.

Normocytic and normochromic anemia with low erythropoietin levels is present in ~ 25% of patients with hereditary transthyretin amyloidosis [14]. Anemia can worsen nOH and should be investigated and treated [19]. Correction of anemia with erythropoietin (25–50 units/kg, subcutaneous, 3 times a week) and iron supplements may be beneficial in patients with nOH [14, 106].

### Non-pharmacologic treatment and patient education

Patients should be aware of the diuretic effects of caffeine and alcohol and avoid sugary beverages (e.g., bottled juices, sodas) because of the the hypotensive effects of high-glycemic index carbohydrates [118]. Fluid intake should be 2–2.5 l/day. Patients should be encouraged to increase salt intake by adding 1–2 teaspoons of salt to a healthy diet. Other patients prefer using 0.5–1.0 g salt tablets although they can cause abdominal discomfort. In patients with nOH, drinking 0.5 l of water produces a marked increase in BP [88]. This can be used as a rescue measure since the pressor effect is quick (peaks in around 30 min) although short-lived.

Symptomatic nOH can quickly lead to an unwillingness to stand up and avoidance of physical activity. In turn, physical immobility worsens OH, leading to a “vicious cycle” of deconditioning [41]. Physical exercise is therefore a key component of the therapeutic regimen, but because physical activity in the standing position can worsen nOH in patients with hereditary transthyretin amyloidosis [81, 110, 122–124], exercise should be performed in the recumbent or sitting position using a recumbent stationary bicycle or rowing machine. The exception is exercise in a pool as the hydrostatic pressure of water allows upright exercise without hypotension [111]. Patients should be taught specific physical countermaneuvers [140]. Eating results in blood pooling within the splanchnic circulation, and patients can become severely hypotensive within 2 h of eating (i.e., postprandial hypotension), particularly after carbohydrate-rich meals [42, 62, 75, 105]. Eating smaller, more frequent meals and reducing carbohydrates can improve postprandial hypotension. Alcohol is also a vasodilator and should be reserved for the evening, prior to going to bed.

High-waist compression stockings producing at least 15–20 mmHg of pressure can increase BP by augmenting venous return [36]. Patients with painful neuropathy struggle to wear the stockings, which limits their usefulness in everyday life. Elastic abdominal binders are a good alternative [40, 121].

### Pharmacologic management

Even after non-pharmacologic methods have been properly implemented, many patients still require pharmacologic treatment to improve symptomatic nOH. Two complementary strategies are commonly used: (1) expanding intravascular volume with fludrocortisone and (2) increasing peripheral vascular resistance with midodrine or droxidopa. Selection of one or the other or both depends on the specific features and needs of each patient as well as the degree of peripheral sympathetic denervation.

#### Fludrocortisone

Fludrocortisone (9α-fluorocortisol) is a synthetic mineralocorticoid that increases BP by at least two mechanisms: it increases renal sodium and water re-absorption, thus expanding intravascular volume, and also enhances the pressor responsiveness to endogenous catecholamine and pressor drugs [26]. Fludrocortisone is extensively used in patients with nOH [103, 104]. There are no specific controlled studies of fludrocortisone for nOH in patients with hereditary transthyretin amyloidosis although its use has been anecdotally described [20, 89, 109]. Fludrocortisone exacerbates supine hypertension and target organ damage (left ventricular hypertrophy and renal failure) and may increase the risk of all-cause hospitalization [50]. It should be used with extreme caution—or not at all—in patients with amyloid cardiomyopathy [39]. Additional, frequent adverse events include hypokalemia and ankle edema [26, 98]. To reduce the risk of hypokalemia, patients taking fludrocortisone should be instructed to eat potassium-rich foods or potassium chloride supplements 10–20 mEq/day. Fludrocortisone dosage should not exceed 0.2 mg/day. Higher dosages are rarely more effective but intensify adverse events. Appreciable clinical improvements usually require ~ 7 days of treatment.

#### Midodrine

Midodrine is an oral α_1_-adrenoreceptor agonist that induces vasoconstriction and increases BP [61, 84, 125, 141]. Midodrine is extensively used in patients with nOH [103, 104]. There are no specific controlled studies of midodrine for nOH in patients with hereditary transthyretin amyloidosis although its use has been anecdotally described [82, 89, 109, 112]. Midodrine raises BP in the standing, sitting and supine positions and its pressor effect is noticeable ~ 30–45 min after consumption, reaching a maximum after ~ 1 h, and persists for a total of 2–3 h. Treatment should begin with a 2.5 or 5 mg dose, which can then be increased up to 10 mg to be taken up to three times a day. Supine hypertension is common; hence, patients should not take midodrine < 3–4 h before bedtime. Other adverse events owing to activation of α1-adrenergic receptors are piloerection (“goosebumps”), itching of the scalp and urinary retention. Midodrine has no effect on heart rate as it does not activate β-adrenoreceptors and, given its poor diffusion across the blood-brain barrier, has no CNS adverse effects [91].

#### Droxidopa (Northera)

Droxidopa (l-threo-3,4-dihydroxyphenyl-serine, l-DOPS) is an oral synthetic amino acid that, once absorbed, is converted to norepinephrine by the enzyme aromatic amino-acid decarboxylase (AAAD) [68]. Early studies in Japan showed that droxidopa increased norepinephrine levels and blood pressure when standing and improved orthostatic tolerance in patients with nOH caused by hereditary transthyretin amyloidosis [7, 129–131]. Consequently, droxidopa was specifically approved in Japan in 1989 for the treatment of nOH in hereditary amyloidosis as well as Parkinson disease and multiple system atrophy. In the US, the Food and Drug Administration (FDA) approved droxidopa in 2014 for the treatment of symptomatic nOH associated with Parkinson disease, multiple system atrophy, pure autonomic failure and non-diabetic autonomic neuropathy, which includes hereditary transthyretin amyloidosis among other causes of autonomic neuropathy [38, 54, 64–66]. Droxidopa is not approved in Europe.

Extensive clinical experience shows that droxidopa is safe and well tolerated [28, 47, 48, 51, 52, 63, 76, 92, 139], even in severely ill patients [90]. Peak plasma concentrations of droxidopa are reached ~ 3 h after oral administration. The dosage used in clinical trials was 100–600 mg three times/day, although clinical experience indicates that the dosage should be tailored to each patient’s needs considering the periods of time when he/she is going to be active or inactive [47, 51, 68]. Because the pressor effect of droxidopa varies among patients, a titration procedure supervised by a clinician is highly recommended [103]. ABPM is useful to evaluate the patient’s BP profile before and after initiating treatment with droxidopa [67].

## Effect of disease-modifying amyloidosis treatment on OH

### Liver transplantation

Because the liver produces the vast majority of mutated transthyretin, orthotopic liver transplantation was widely used to stop the production of transthyretin for hereditary transthyretin amyloidosis before the availability of transthyretin stabilizers (diflunisal, tafamidis) and RNA interference agents (patisiran, inotersen). Longitudinal evaluation of patients after transplantation showed that progression of autonomic neuropathy (i.e., cardiac sympathetic denervation) was arrested, resulting in significant improvement in symptoms of nOH [4, 13, 17, 32, 35, 59, 117, 126].

### TTR stabilizers

Diflunisal, a nonsteroidal anti-inflammatory drug available in most countries, is effective to stabilize circulating transthyretin tetramers, inhibiting the release of the transthyretin monomer required for amyloid deposition [94, 116]. A 2-year double-blind placebo-controlled trial showed that diflunisal reduced the rate of progression of neurologic impairment and preserved quality of life in patients with hereditary transthyretin amyloidosis [18]. The main outcome measure was the Neuropathy Impairment Score plus seven nerve tests (NIS + 7), which assesses motor and sensory signs, but does not include autonomic items. Secondary outcome measures included the Kumamoto score, a neurologic scale of motor, sensory and autonomic function [135]. However, changes in the autonomic domains were not specifically reported [18]. The effect of diflunisal on autonomic function was specifically evaluated in six patients with late-onset Val20Met variant showing complete abatement of symptoms of nOH, accompanied by increased cardiac sympathetic innervation [133], suggesting that diflunisal might be useful to specifically slow the progression of autonomic neuropathy.

Tafamidis, a TTR stabilizer, was approved by the European Medicines Agency in 2012 and Japan in 2013 for the treatment of transthyretin amyloid polyneuropathy. The US FDA had not approved tafamidis at the time of publishing this article. The effects of tafamidis on nOH and other autonomic markers were not reported in the clinical trials studying its effects on neuropathy or cardiomyopathy [29, 30, 87]. In a study performed in 29 patients with late-onset Val20Met variant receiving tafamidis, [85], autonomic scores worsened in 6 (21%) with 2 patients developing OH [85]. Overall, the effect of tafamidis on nOH remains unclear.

### RNA interference agents

Two phase-3 clinical trials with patisiran and inotersen, newly developed RNA-based antisense therapies approved by the US FDA and the European Medicines Agency in 2018, showed that blocking the production of transthyretin improves the polyneuropathy and quality of life of patients with hereditary transthyretin amyloidosis [1, 16]. Both trials used the modified NIS + 7 (mNIS + 7) as primary outcome measure. In contrast to the NIS + 7, the mNIS + 7 includes BP orthostatic measurements, although this contributes to only 2 out of 304 points of the total score. The patisiran trial reported significant improvements in the BP subcomponent of the mNIS + 7, although this was sparsely reported in a qualitative manner (range: 0–2, where 0 denotes no BP drop, 1 denotes a BP drop ≤ 30 mmHg, and 2 denotes a BP drop ≥ 30 mmHg) [1]. The inotersen trial did not report any information on BP changes [16]. A specific study on whether RNA interference agents abate the orthostatic drop in BP and improve symptoms of nOH in patients with hereditary transthyretin amyloidosis is warranted.

## Conclusion

Orthostatic hypotension is a prominent and disabling manifestation of autonomic dysfunction in patients with hereditary transthyretin (TTR) amyloidosis affecting an estimated 40–60% of patients, reducing quality of life. Orthostatic hypotension in patients with hereditary transthyretin amyloidosis can be a consequence of heart failure due to amyloid cardiomyopathy or volume depletion due to diarrhea or drug effects. When none of these circumstances are apparent, orthostatic hypotension is usually neurogenic, i.e., caused by impaired norepinephrine release from sympathetic postganglionic neurons, because of neuronal amyloid fibril deposition. When recognized, orthostatic hypotension can be treated. Discontinuation of potentially causative/aggravating drugs, patient education and non-pharmacologic approaches are valuable and should be applied first. Droxidopa (Northera^®^), a synthetic norepinephrine precursor, has shown efficacy in controlled trials of neurogenic orthostatic hypotension in patients with hereditary TTR amyloidosis and is now approved in the US and Asia. Novel disease-modifying treatments such as transthyretin stabilizers (diflunisal, tafamidis) and RNA interference agents (patisiran, inotersen) may have an impact on the natural history of nOH in patients with hereditary TTR amyloidosis, although dedicated studies are yet to be performed.

## References

[1] Adams D, Gonzalez-Duarte A, O’Riordan WD, Yang C-C, Ueda M, Kristen AV, Tournev I, Schmidt HH, Coelho T, Berk JL, Lin K-P, Vita G, Attarian S, Planté-Bordeneuve V, Mezei MM, Campistol JM, Buades J, Brannagan TH, Kim BJ, Oh J, Parman Y, Sekijima Y, Hawkins PN, Solomon SD, Polydefkis M, Dyck PJ, Gandhi PJ, Goyal S, Chen J, Strahs AL, Nochur SV, Sweetser MT, Garg PP, Vaishnaw AK, Gollob JA, Suhr OB (2018). Patisiran, an RNAi therapeutic, for hereditary transthyretin amyloidosis. N Engl J Med.

[2] Adams D, Suhr OB, Hund E, Obici L, Tournev I, Campistol JM, Slama MS, Hazenberg BP, Coelho T, European Network for T-F (2016). First European consensus for diagnosis, management, and treatment of transthyretin familial amyloid polyneuropathy. Curr Opin Neurol.

[3] Algalarrondo V, Antonini T, Theaudin M, Chemla D, Benmalek A, Lacroix C, Castaing D, Cauquil C, Dinanian S, Eliahou L, Samuel D, Adams D, Le Guludec D, Slama MS, Rouzet F (2016). Cardiac dysautonomia predicts long-term survival in hereditary transthyretin amyloidosis after liver transplantation. JACC Cardiovasc Imaging.

[4] Algalarrondo V, Antonini T, Theaudin M, Ducot B, Lozeron P, Chemla D, Benmalek A, Lacroix C, Azoulay D, Castaing D, Cauquil C, Rouzet F, Dinanian S, Eliahou L, Le Guludec D, Samuel D, Slama MS, Adams D (2015). Prediction of long-term survival after liver transplantation for familial transthyretin amyloidosis. J Am Coll Cardiol.

[5] Algalarrondo V, Eliahou L, Thierry I, Bouzeman A, Dasoveanu M, Sebag C, Moubarak G, Le Guludec D, Samuel D, Adams D, Dinanian S, Slama MS (2012). Circadian rhythm of blood pressure reflects the severity of cardiac impairment in familial amyloid polyneuropathy. Arch Cardiovasc Dis.

[6] Ando Y, Araki S, Shimoda O, Kano T (1992). Role of autonomic nerve functions in patients with familial amyloidotic polyneuropathy as analyzed by laser Doppler flowmetry, capsule hydrograph, and cardiographic R–R interval. Muscle Nerve.

[7] Ando Y, Tanaka Y, Yamashita T, Tashima K, Sakashita N, Nakamura M, Uchino M, Ando M (1995). Familial amyloidotic polyneuropathy (FAP) type I and the therapies. Rinsho shinkeigaku.

[8] Andrade C (1952). A peculiar form of peripheral neuropathy; familiar atypical generalized amyloidosis with special involvement of the peripheral nerves. Brain.

[9] Araki S, Mawatari S, Ohta M, Nakajima A, Kuroiwa Y (1968). Polyneuritic amyloidosis in a Japanese family. Arch Neurol.

[10] Araki S, Yi S (2000). Pathology of familial amyloidotic polyneuropathy with TTR met 30 in Kumamoto, Japan. Neuropathology.

[11] Arnold AC, Biaggioni I (2012). Management approaches to hypertension in autonomic failure. Curr Opin Nephrol Hypertens.

[12] Asakura K, Yanai S, Nakamura S, Kawaski K, Eizuka M, Ishida K, Sugai T, Ueda M, Yamashita T, Ando Y, Matsumoto T (2016). Endoscopic findings of small-bowel lesions in familial amyloid polyneuropathy: a case report. Medicine.

[13] Azevedo Coutinho MDC, Cortez-Dias N, Cantinho G, Conceicao I, Guimaraes T, Lima da Silva G, Nobre Menezes M, Francisco AR, Placido R, Pinto FJ (2017). Progression of myocardial sympathetic denervation assessed by (123)I-MIBG imaging in familial amyloid polyneuropathy and the effect of liver transplantation. Rev Port Cardiol.

[14] Beirao I, Lobato L, Moreira L, Mp Costa P, Fonseca I, Cabrita A, Porto G (2008). Long-term treatment of anemia with recombinant human erythropoietin in familial amyloidosis TTR V30M. Amyloid.

[15] Benson MD, Turpin JC, Lucotte G, Zeldenrust S, LeChevalier B, Benson MD (1993). A transthyretin variant (alanine 71) associated with familial amyloidotic polyneuropathy in a French family. J Med Genet.

[16] Benson MD, Waddington-Cruz M, Berk JL, Polydefkis M, Dyck PJ, Wang AK, Planté-Bordeneuve V, Barroso FA, Merlini G, Obici L, Scheinberg M, Brannagan TH, Litchy WJ, Whelan C, Drachman BM, Adams D, Heitner SB, Conceição I, Schmidt HH, Vita G, Campistol JM, Gamez J, Gorevic PD, Gane E, Shah AM, Solomon SD, Monia BP, Hughes SG, Kwoh TJ, McEvoy BW, Jung SW, Baker BF, Ackermann EJ, Gertz MA, Coelho T (2018). Inotersen treatment for patients with hereditary transthyretin amyloidosis. N Engl J Med.

[17] Bergethon PR, Sabin TD, Lewis D, Simms RW, Cohen AS, Skinner M (1996). Improvement in the polyneuropathy associated with familial amyloid polyneuropathy after liver transplantation. Neurology.

[18] Berk JL, Suhr OB, Obici L, Sekijima Y, Zeldenrust SR, Yamashita T, Heneghan MA, Gorevic PD, Litchy WJ, Wiesman JF, Nordh E, Corato M, Lozza A, Cortese A, Robinson-Papp J, Colton T, Rybin DV, Bisbee AB, Ando Y, Ikeda S, Seldin DC, Merlini G, Skinner M, Kelly JW, Dyck PJ, Diflunisal Trial C (2013). Repurposing diflunisal for familial amyloid polyneuropathy: a randomized clinical trial. J Am Med Assoc.

[19] Biaggioni I, Robertson D, Krantz S, Jones M, Haile V (1994). The anemia of primary autonomic failure and its reversal with recombinant erythropoietin. Ann Intern Med.

[20] Brett M, Persey MR, Reilly MM, Revesz T, Booth DR, Booth SE, Hawkins PN, Pepys MB, Morgan-Hughes JA (1999). Transthyretin Leu12Pro is associated with systemic, neuropathic and leptomeningeal amyloidosis. Brain.

[21] Cappellari M, Cavallaro T, Ferrarini M, Cabrini I, Taioli F, Ferrari S, Merlini G, Obici L, Briani C, Fabrizi GM (2011). Variable presentations of TTR-related familial amyloid polyneuropathy in seventeen patients. J Peripher Nerv Syst.

[22] Carvalho MJ, van Den Meiracker AH, Boomsma F, Lima M, Freitas J, Veld AJ, Falcao De Freitas A (2000). Diurnal blood pressure variation in progressive autonomic failure. Hypertension.

[23] Castano A, Drachman BM, Judge D, Maurer MS (2015). Natural history and therapy of TTR-cardiac amyloidosis: emerging disease-modifying therapies from organ transplantation to stabilizer and silencer drugs. Heart Fail Rev.

[24] Chao CC, Huang CM, Chiang HH, Luo KR, Kan HW, Yang NC, Chiang H, Lin WM, Lai SM, Lee MJ, Shun CT, Hsieh ST (2015). Sudomotor innervation in transthyretin amyloid neuropathy: pathology and functional correlates. Ann Neurol.

[25] Cho HJ, Yoon JY, Bae MH, Lee JH, Yang DH, Park HS, Cho Y, Chae SC, Jun JE (2012). Familial transthyretin amyloidosis with variant Asp38Ala presenting with orthostatic hypotension and chronic diarrhea. J Cardiovasc Ultrasound.

[26] Chobanian AV, Volicer L, Tifft CP, Gavras H, Liang CS, Faxon D (1979). Mineralocorticoid-induced hypertension in patients with orthostatic hypotension. N Engl J Med.

[27] Chou CT, Lee CC, Chang DM, Buxbaum JN, Jacobson DR (1997). Familial amyloidosis in one Chinese family: clinical, immunological, and molecular genetic analysis. J Intern Med.

[28] Claassen D, Lew M (2017). Initiating droxidopa for neurogenic orthostatic hypotension in a patient with Parkinson disease. Clin Auton Res.

[29] Coelho T, Maia LF, da Silva AM, Cruz MW, Planté-Bordeneuve V, Suhr OB, Conceiçao I, Schmidt HH-J, Trigo P, Kelly JW, Labaudinière R, Chan J, Packman J, Grogan DR (2013). Long-term effects of tafamidis for the treatment of transthyretin familial amyloid polyneuropathy. J Neurol.

[30] Coelho T, Maia LF, Martins da Silva A, Waddington Cruz M, Plante-Bordeneuve V, Lozeron P, Suhr OB, Campistol JM, Conceicao IM, Schmidt HH, Trigo P, Kelly JW, Labaudiniere R, Chan J, Packman J, Wilson A, Grogan DR (2012). Tafamidis for transthyretin familial amyloid polyneuropathy: a randomized, controlled trial. Neurology.

[31] Coutinho MC, Cortez-Dias N, Cantinho G, Conceicao I, Oliveira A, Bordalo AS, Goncalves S, Almeida AG, de Carvalho M, Diogo AN (2013). Reduced myocardial 123-iodine metaiodobenzylguanidine uptake: a prognostic marker in familial amyloid polyneuropathy. Circ Cardiovasc Imaging.

[32] Dai WC, Chan SC, Chok KS, Cheung TT, Sharr WW, Chan AC, Fung JY, Tsang SH, Fan ST, Lo CM (2012). Single-centre experience of liver transplantation for familial amyloidotic polyneuropathy of non-Val30Met variants in Chinese patients. Amyloid.

[33] Davies DR, Smith SE (1999). Pupil abnormality in amyloidosis with autonomic neuropathy. J Neurol Neurosurg Psychiatry.

[34] Delahaye N, Dinanian S, Slama MS, Mzabi H, Samuel D, Adams D, Merlet P, Le Guludec D (1999). Cardiac sympathetic denervation in familial amyloid polyneuropathy assessed by iodine-123 metaiodobenzylguanidine scintigraphy and heart rate variability. Eur J Nucl Med.

[35] Delahaye N, Rouzet F, Sarda L, Tamas C, Dinanian S, Plante-Bordeneuve V, Adams D, Samuel D, Merlet P, Syrota A, Slama MS, Le Guludec D (2006). Impact of liver transplantation on cardiac autonomic denervation in familial amyloid polyneuropathy. Medicine.

[36] Diedrich A, Biaggioni I (2004). Segmental orthostatic fluid shifts. Clinical Auton Res.

[37] Ducla-Soares J, Alves MM, Carvalho M, Povoa P, Conceicao I, Sales Luis ML (1994). Correlation between clinical, electromyographic and dysautonomic evolution of familial amyloidotic polyneuropathy of the Portuguese type. Acta Neurol Scand.

[38] Elgebaly A, Abdelazeim B, Mattar O, Gadelkarim M, Salah R, Negida A (2016). Meta-analysis of the safety and efficacy of droxidopa for neurogenic orthostatic hypotension. Clin Auton Res.

[39] Falk RH, Dubrey SW, Gertz MA, Rajkumar SV (2010). Amyloid Heart Disease. Amyloidosis: diagnosis and treatment.

[40] Fanciulli A, Goebel G, Metzler B, Sprenger F, Poewe W, Wenning GK, Seppi K (2016). Elastic abdominal binders attenuate orthostatic hypotension in Parkinson’s disease. Mov Disord Clin Pract.

[41] Freeman R (2008). Clinical practice. Neurogenic orthostatic hypotension. N Engl J Med.

[42] Freeman R, Wieling W, Axelrod FB, Benditt DG, Benarroch E, Biaggioni I, Cheshire WP, Chelimsky T, Cortelli P, Gibbons CH, Goldstein DS, Hainsworth R, Hilz MJ, Jacob G, Kaufmann H, Jordan J, Lipsitz LA, Levine BD, Low PA, Mathias C, Raj SR, Robertson D, Sandroni P, Schatz I, Schondorff R, Stewart JM, van Dijk JG (2011). Consensus statement on the definition of orthostatic hypotension, neurally mediated syncope and the postural tachycardia syndrome. Clin Auton Res.

[43] Fujitake J, Horii K, Tatsuoka Y, Funauchi M, Saida K (1991). Two brother cases of late-onset familial amyloidotic polyneuropathy in Kyoto. Rinsho Shinkeigaku.

[44] Gibbons CH, Schmidt P, Biaggioni I, Frazier-Mills C, Freeman R, Isaacson S, Karabin B, Kuritzky L, Lew M, Low P, Mehdirad A, Raj SR, Vernino S, Kaufmann H (2017). The recommendations of a consensus panel for the screening, diagnosis, and treatment of neurogenic orthostatic hypotension and associated supine hypertension. J Neurol.

[45] Gonzalez-Duarte A (2018). Autonomic involvement in hereditary transthyretin amyloidosis (hATTR amyloidosis). Clin Auton Res.

[46] Gonzalez-Duarte A, Mundayat R, Shapiro B (2017). Assessing the onset and characteristics of orthostatic hypotension in patients with transthyretin amyloidosis from the transthyretin amyloidosis outcomes survey (THAOS). J Neurol Sci.

[47] Goodman BP, Claassen D, Mehdirad A (2017). Adjusting droxidopa for neurogenic orthostatic hypotension in a patient with Parkinson disease. Clin Auton Res.

[48] Goodman BP, Gupta F (2017). Defining successful treatment of neurogenic orthostatic hypotension with droxidopa in a patient with multiple system atrophy. Clin Auton Res.

[49] Grazi GL, Cescon M, Salvi F, Ercolani G, Ravaioli M, Arpesella G, Magelli C, Grigioni F, Cavallari A (2003). Combined heart and liver transplantation for familial amyloidotic neuropathy: considerations from the hepatic point of view. Liver Transpl.

[50] Grijalva CG, Biaggioni I, Griffin MR, Shibao CA (2017). Fludrocortisone is associated with a higher risk of all-cause hospitalizations compared with midodrine in patients with orthostatic hypotension. J Am Heart Assoc.

[51] Gupta F, Karabin B, Mehdirad A (2017). Titrating droxidopa to maximize symptomatic benefit in a patient with Parkinson disease and neurogenic orthostatic hypotension. Clin Auton Res.

[52] Gupta F, Kremens D, Vernino S, Karabin B (2017). Managing neurogenic orthostatic hypotension in a patient presenting with pure autonomic failure who later developed Parkinson disease. Clin Auton Res.

[53] Haagsma EB, Scheffer H, Altland K, De Jager AE, Hazenberg BP (2000). Transthyretin Val71Ala mutation in a Dutch family with familial amyloidotic polyneuropathy. Amyloid.

[54] Hauser RA, Isaacson S, Lisk JP, Hewitt LA, Rowse G (2015). Droxidopa for the short-term treatment of symptomatic neurogenic orthostatic hypotension in Parkinson’s disease (nOH306B). Mov Disord.

[55] Hirakawa K, Takashio S, Marume K, Yamamoto M, Hanatani S, Yamamoto E, Sakamoto K, Izumiya Y, Kaikita K, Oda S, Utsunomiya D, Shiraishi S, Ueda M, Yamashita T, Yamashita Y, Ando Y, Tsujita K (2018). Non-Val30Met mutation, septal hypertrophy, and cardiac denervation in patients with mutant transthyretin amyloidosis. ESC Heart Fail.

[56] Hirayama M, Hakusui S, Koike Y, Ito K, Kato T, Ikeda M, Hasegawa Y, Takahashi A (1995). A scintigraphical qualitative analysis of peripheral vascular sympathetic function with meta-[123I]iodobenzylguanidine in neurological patients with autonomic failure. J Auton Nerv Syst.

[57] Hsieh ST (2011). Amyloid neuropathy with transthyretin mutations: overview and unique Ala97Ser in Taiwan. Acta Neurol Taiwan.

[58] Hsu HC, Liao MF, Hsu JL, Lo AL, Kuo HC, Lyu RK, Wu VC, Wang CW, Ro LS (2017). Phenotypic expressions of hereditary transthyretin Ala97Ser related amyloidosis (ATTR) in Taiwanese. BMC Neurol.

[59] Ikeda S, Takei Y, Yanagisawa N, Matsunami H, Hashikura Y, Ikegami T, Kawasaki S (1997). Peripheral nerves regenerated in familial amyloid polyneuropathy after liver transplantation. Ann Intern Med.

[60] Jacobson DR, McFarlin DE, Kane I, Buxbaum JN (1992). Transthyretin Pro55, a variant associated with early-onset, aggressive, diffuse amyloidosis with cardiac and neurologic involvement. Hum Genet.

[61] Jankovic J, Gilden JL, Hiner BC, Kaufmann H, Brown DC, Coghlan CH, Rubin M, Fouad-Tarazi FM (1993). Neurogenic orthostatic hypotension: a double-blind, placebo-controlled study with midodrine. Am J Med.

[62] Jansen RW, Lipsitz LA (1995). Postprandial hypotension: epidemiology, pathophysiology, and clinical management. Ann Intern Med.

[63] Kaufmann H (2017). Droxidopa for symptomatic neurogenic orthostatic hypotension: what can we learn?. Clin Auton Res.

[64] Kaufmann H, Biaggioni I (2003). Autonomic failure in neurodegenerative disorders. Semin Neurol.

[65] Kaufmann H, Freeman R, Biaggioni I, Low P, Pedder S, Hewitt LA, Mauney J, Feirtag M, Mathias CJ, Investigators NOH (2014). Droxidopa for neurogenic orthostatic hypotension: a randomized, placebo-controlled, phase 3 trial. Neurology.

[66] Kaufmann H, Malamut R, Norcliffe-Kaufmann L, Rosa K, Freeman R (2012). The Orthostatic Hypotension Questionnaire (OHQ): validation of a novel symptom assessment scale. Clin Auton Res.

[67] Kaufmann H, Norcliffe-Kaufmann L, Hewitt LA, Rowse GJ, White WB (2016). Effects of the novel norepinephrine prodrug, droxidopa, on ambulatory blood pressure in patients with neurogenic orthostatic hypotension. J Am Soc Hypertens.

[68] Kaufmann H, Norcliffe-Kaufmann L, Palma JA (2015). Droxidopa in neurogenic orthostatic hypotension. Expert Rev Cardiovasc Ther.

[69] Kim DH, Zeldenrust SR, Low PA, Dyck PJ (2009). Quantitative sensation and autonomic test abnormalities in transthyretin amyloidosis polyneuropathy. Muscle Nerve.

[70] Koike H, Misu K, Ikeda S, Ando Y, Nakazato M, Ando E, Yamamoto M, Hattori N, Sobue G, Study Group for Hereditary Neuropathy in J (2002). Type I (transthyretin Met30) familial amyloid polyneuropathy in Japan: early- vs late-onset form. Arch Neurol.

[71] Koike H, Misu K, Sugiura M, Iijima M, Mori K, Yamamoto M, Hattori N, Mukai E, Ando Y, Ikeda S, Sobue G (2004). Pathology of early- vs late-onset TTR Met30 familial amyloid polyneuropathy. Neurology.

[72] Koike H, Nakamura T, Hashizume A, Nishi R, Ikeda S, Kawagashira Y, Iijima M, Katsuno M, Sobue G (2017). Cardiac and peripheral vasomotor autonomic functions in late-onset transthyretin Val30Met familial amyloid polyneuropathy. J Neurol.

[73] Koike H, Nakamura T, Nishi R, Ikeda S, Kawagashira Y, Iijima M, Katsuno M, Sobue G (2018). Widespread cardiac and vasomotor autonomic dysfunction in non-Val30Met hereditary transthyretin amyloidosis. Intern Med.

[74] Koike H, Tanaka F, Hashimoto R, Tomita M, Kawagashira Y, Iijima M, Fujitake J, Kawanami T, Kato T, Yamamoto M, Sobue G (2012). Natural history of transthyretin Val30Met familial amyloid polyneuropathy: analysis of late-onset cases from non-endemic areas. J Neurol Neurosurg Psychiatry.

[75] Kooner JS, Raimbach S, Watson L, Bannister R, Peart S, Mathias CJ (1989). Relationship between splanchnic vasodilation and postprandial hypotension in patients with primary autonomic failure. J Hypertens Suppl.

[76] Kremens D, Lew M, Claassen D, Goodman BP (2017). Adding droxidopa to fludrocortisone or midodrine in a patient with neurogenic orthostatic hypotension and Parkinson disease. Clin Auton Res.

[77] Lahrmann H, Cortelli P, Hilz M, Mathias CJ, Struhal W, Tassinari M (2006). EFNS guidelines on the diagnosis and management of orthostatic hypotension. Eur J Neurol.

[78] Liao MF, Chang HS (2013). A novel variant mutation of transthyretin Ile73Val-related amyloidotic polyneuropathy in Taiwanese. Acta Neurol Taiwan.

[79] Liu JY, Guo YJ, Zhou CK, Ye YQ, Feng JQ, Yin F, Jiang XM (2011). Clinical and histopathological features of familial amyloidotic polyneuropathy with transthyretin Val30Ala in a Chinese family. J Neurol Sci.

[80] Loavenbruck AJ, Singer W, Mauermann ML, Sandroni P, PJ BD, Gertz M, Klein CJ, Low PA (2016). Transthyretin amyloid neuropathy has earlier neural involvement but better prognosis than primary amyloid counterpart: an answer to the paradox?. Ann Neurol.

[81] Low DA, Vichayanrat E, Iodice V, Mathias CJ (2014). Exercise hemodynamics in Parkinson’s disease and autonomic dysfunction. Parkinsonism Relat Disord.

[82] Low PA (1998). Autonomic neuropathies. Curr Opin Neurol.

[83] Low PA, Dyck PJ, Okazaki H, Kyle R, Fealey RD (1981). The splanchnic autonomic outflow in amyloid neuropathy and Tangier disease. Neurology.

[84] Low PA, Gilden JL, Freeman R, Sheng KN, McElligott MA (1997). Efficacy of midodrine vs placebo in neurogenic orthostatic hypotension. A randomized, double-blind multicenter study. Midodrine Study Group. J Am Med Assoc.

[85] Lozeron P, Theaudin M, Mincheva Z, Ducot B, Lacroix C, Adams D, French Network for FAP (2013). Effect on disability and safety of Tafamidis in late onset of Met30 transthyretin familial amyloid polyneuropathy. Eur J Neurol.

[86] Mariani LL, Lozeron P, Theaudin M, Mincheva Z, Signate A, Ducot B, Algalarrondo V, Denier C, Adam C, Nicolas G, Samuel D, Slama MS, Lacroix C, Misrahi M, Adams D, French Familial Amyloid Polyneuropathies Network Study G (2015). Genotype-phenotype correlation and course of transthyretin familial amyloid polyneuropathies in France. Ann Neurol.

[87] Maurer MS, Schwartz JH, Gundapaneni B, Elliott PM, Merlini G, Waddington-Cruz M, Kristen AV, Grogan M, Witteles R, Damy T, Drachman BM, Shah SJ, Hanna M, Judge DP, Barsdorf AI, Huber P, Patterson TA, Riley S, Schumacher J, Stewart M, Sultan MB, Rapezzi C (2018). Tafamidis treatment for patients with transthyretin amyloid cardiomyopathy. N Engl J Med.

[88] May M, Jordan J (2011). The osmopressor response to water drinking. Am J Physiol Regul Integr Comp Physiol.

[89] Mazzeo A, Russo M, Di Bella G, Minutoli F, Stancanelli C, Gentile L, Baldari S, Carerj S, Toscano A, Vita G (2015). Transthyretin-related familial amyloid polyneuropathy (TTR-FAP): a single-center experience in Sicily, an Italian Endemic Area. J Neuromuscul Dis.

[90] McDonell Katherine E., Preheim Brock A., Diedrich Andre’, Muldowney James A. S., Peltier Amanda C., Robertson David, Biaggioni Italo, Shibao Cyndya A. (2019). Initiation of droxidopa during hospital admission for management of refractory neurogenic orthostatic hypotension in severely ill patients. The Journal of Clinical Hypertension.

[91] McTavish D, Goa KL (1989). Midodrine. A review of its pharmacological properties and therapeutic use in orthostatic hypotension and secondary hypotensive disorders. Drugs.

[92] Mehdirad A, Karabin B, Gupta F (2017). Managing neurogenic orthostatic hypotension with droxidopa in a patient with Parkinson disease, atrial fibrillation, and hypertension. Clin Auton Res.

[93] Mesquita ET, Jorge AJL, Souza CVJ, Andrade TR (2017). Cardiac amyloidosis and its new clinical phenotype: heart failure with preserved ejection fraction. Arq Bras Cardiol.

[94] Miller SR, Sekijima Y, Kelly JW (2004). Native state stabilization by NSAIDs inhibits transthyretin amyloidogenesis from the most common familial disease variants. Lab Invest.

[95] Munar-Ques M, Masjuan J, Coelho T, Moreira P, Viader-Farre C, Saraiva MJ (2007). Familial amyloid polyneuropathy associated with TTRSer50Arg mutation in two Iberian families presenting a novel single base change in the mutant gene. Amyloid.

[96] Nagasaka T, Togashi S, Watanabe H, Iida H, Nagasaka K, Nakamura Y, Miwa M, Kobayashi F, Shindo K, Shiozawa Z (2009). Clinical and histopathological features of progressive-type familial amyloidotic polyneuropathy with TTR Lys54. J Neurol Sci.

[97] Nakata T, Shimamoto K, Yonekura S, Kobayashi N, Sugiyama T, Imai K, Iimura O (1995). Cardiac sympathetic denervation in transthyretin-related familial amyloidotic polyneuropathy: detection with iodine-123-MIBG. J Nucl Med.

[98] Norcliffe-Kaufmann L, Axelrod FB, Kaufmann H (2013). Developmental abnormalities, blood pressure variability and renal disease in Riley Day syndrome. J Hum Hypertens.

[99] Norcliffe-Kaufmann L, Kaufmann H (2014). Is ambulatory blood pressure monitoring useful in patients with chronic autonomic failure?. Clin Auton Res.

[100] Norcliffe-Kaufmann L, Kaufmann H, Palma JA, Shibao CA, Biaggioni I, Peltier AC, Singer W, Low PA, Goldstein DS, Gibbons CH, Freeman R, Robertson D, Autonomic Disorders C (2018). Orthostatic heart rate changes in patients with autonomic failure caused by neurodegenerative synucleinopathies. Ann Neurol.

[101] Obayashi K, Ando Y (2012). Focus on autonomic dysfunction in familial amyloidotic polyneuropathy (FAP). Amyloid.

[102] Palma JA, Carmona-Abellan MM, Barriobero N, Trevino-Peinado C, Garcia-Lopez M, Fernandez-Jarne E, Luquin MR (2013). Is cardiac function impaired in premotor Parkinson’s disease? A retrospective cohort study. Mov Disord.

[103] Palma JA, Kaufmann H (2017). Epidemiology, diagnosis, and management of neurogenic orthostatic hypotension. Mov Disord Clin Pract.

[104] Palma JA, Kaufmann H (2018). Treatment of autonomic dysfunction in Parkinson disease and other synucleinopathies. Mov Disord.

[105] Pavelic A, Krbot Skoric M, Crnosija L, Habek M (2017). Postprandial hypotension in neurological disorders: systematic review and meta-analysis. Clin Auton Res.

[106] Perera R, Isola L, Kaufmann H (1995). Effect of recombinant erythropoietin on anemia and orthostatic hypotension in primary autonomic failure. Clin Auton Res.

[107] Plante-Bordeneuve V (2014). Update in the diagnosis and management of transthyretin familial amyloid polyneuropathy. J Neurol.

[108] Plante-Bordeneuve V, Ferreira A, Lalu T, Zaros C, Lacroix C, Adams D, Said G (2007). Diagnostic pitfalls in sporadic transthyretin familial amyloid polyneuropathy (TTR-FAP). Neurology.

[109] Planté-Bordeneuve V, Kerschen P (2013) Transthyretin familial amyloid polyneuropathy. Handb Clin Neurol 115:643–658 10.1016/B978-0-444-52902-2.00038-2 23931808

[110] Puvi-Rajasingham S, Smith GD, Akinola A, Mathias CJ (1997). Abnormal regional blood flow responses during and after exercise in human sympathetic denervation. J Physiol.

[111] Rowell LB (1986). Human circulation: regulation during physical stress.

[112] Russo M, Vita GL, Stancanelli C, Mazzeo A, Vita G, Messina S (2016). Parenteral nutrition improves nutritional status, autonomic symptoms and quality of life in transthyretin amyloid polyneuropathy. Neuromuscul Disord.

[113] Sakashita N, Ando Y, Obayashi K, Terazaki H, Yamashita T, Takei M, Kinjo M, Takahashi K (2000). Familial amyloidotic polyneuropathy (ATTR Ser50Ile): the first autopsy case report. Virchows Arch.

[114] Salhi H, Lefaucheur JP, Gorram F, Rappeneau S, Funalot B, Fanen P, Coste B, Damy T, Planté-Bordeneuve V (2015). Phenotypic spectrum and management of 25 patients ATTR Val122Ile (P5.073). Neurology.

[115] Salvi F, Scaglione C, Michelucci R, Linke RP, Obici L, Ravani A, Rimessi P, Ferlini A, Meletti S, Cavallaro T, Tassinari CA, Martinelli P (2003). Atypical familial motor neuropathy in patients with mutant TTR Ile68Leu. Amyloid.

[116] Sekijima Y, Dendle MA, Kelly JW (2006). Orally administered diflunisal stabilizes transthyretin against dissociation required for amyloidogenesis. Amyloid.

[117] Sharma P, Perri RE, Sirven JE, Zeldenrust SR, Brandhagen DJ, Rosen CB, Douglas DD, Mulligan DC, Rakela J, Wiesner RH, Balan V (2003). Outcome of liver transplantation for familial amyloidotic polyneuropathy. Liver Transpl.

[118] Shibao C, Gamboa A, Diedrich A, Dossett C, Choi L, Farley G, Biaggioni I (2007). Acarbose, an alpha-glucosidase inhibitor, attenuates postprandial hypotension in autonomic failure. Hypertension.

[119] Shimizu H, Ishikawa K, Kobayashi H, Murakami T, Nakazato M, Miura K, Atsumi T (1996). Familial amyloidotic polyneuropathy with a transthyretin variant (Val30– > Leu). No to shinkei Brain and nerve.

[120] Slart R, Glaudemans A, Hazenberg BPC, Noordzij W (2017). Imaging cardiac innervation in amyloidosis. J Nucl Cardiol.

[121] Smit AA, Wieling W, Fujimura J, Denq JC, Opfer-Gehrking TL, Akarriou M, Karemaker JM, Low PA (2004). Use of lower abdominal compression to combat orthostatic hypotension in patients with autonomic dysfunction. Clin Auton Res.

[122] Smith GD, Mathias CJ (1995). Postural hypotension enhanced by exercise in patients with chronic autonomic failure. QJM.

[123] Smith GD, Watson LP, Mathias CJ (1996). Neurohumoral, peptidergic and biochemical responses to supine exercise in two groups with primary autonomic failure: shy-Drager syndrome/multiple system atrophy and pure autonomic failure. Clin Auton Res.

[124] Smith GD, Watson LP, Mathias CJ (1998). Differing haemodynamic and catecholamine responses to exercise in three groups with peripheralautonomic dysfunction: insulin-dependent diabetes mellitus, familial amyloid polyneuropathy and pure autonomic failure. J Auton Nerv Syst.

[125] Smith W, Wan H, Much D, Robinson AG, Martin P (2016). Clinical benefit of midodrine hydrochloride in symptomatic orthostatic hypotension: a phase 4, double-blind, placebo-controlled, randomized, tilt-table study. Clin Auton Res.

[126] Suhr OB, Holmgren G, Ando Y (1998). Improvement in the polyneuropathy associated with familial amyloid polyneuropathy after liver transplantation. Neurology.

[127] Suhr OB, Svendsen IH, Andersson R, Danielsson A, Holmgren G, Ranlov PJ (2003). Hereditary transthyretin amyloidosis from a Scandinavian perspective. J Intern Med.

[128] Suzuki T, Higa S, Sakoda S, Hayashi A, Yamamura Y, Takaba Y, Nakajima A (1981). Orthostatic hypotension in familial amyloid polyneuropathy: treatment with dl-threo-3,4-dihydroxyphenylserine. Neurology.

[129] Suzuki T, Higa S, Sakoda S, Ueji M, Hayashi A, Takaba Y, Nakajima A (1982). Pharmacokinetic studies of oral l-threo-3,4-dihydroxyphenylserine in normal subjects and patients with familial amyloid polyneuropathy. Eur J Clin Pharmacol.

[130] Suzuki T, Higa S, Tsuge I, Sakoda S, Hayashi A, Yamamura Y, Takaba Y, Nakajima A (1980). Effect of infused l-threo-3,4-dihydroxyphenylserine on adrenergic activity in patients with familial amyloid polyneuropathy. Eur J Clin Pharmacol.

[131] Suzuki T, Sakoda S, Ueji M, Kishimoto S (1985). Mass spectrometric measurements of norepinephrine synthesis in man from infusion of stable isotope-labelled l-threo-3,4-dihydroxyphenylserine. Life Sci.

[132] Takahashi K, Yi S, Kimura Y, Araki S (1991). Familial amyloidotic polyneuropathy type 1 in Kumamoto, Japan: a clinicopathologic, histochemical, immunohistochemical, and ultrastructural study. Hum Pathol.

[133] Takahashi R, Ono K, Shibata S, Nakamura K, Komatsu J, Ikeda Y, Ikeda T, Samuraki M, Sakai K, Iwasa K, Kayano D, Yamada M (2014). Efficacy of diflunisal on autonomic dysfunction of late-onset familial amyloid polyneuropathy (TTR Val30Met) in a Japanese endemic area. J Neurol Sci.

[134] Tanaka M, Hongo M, Kinoshita O, Takabayashi Y, Fujii T, Yazaki Y, Isobe M, Sekiguchi M (1997). Iodine-123 metaiodobenzylguanidine scintigraphic assessment of myocardial sympathetic innervation in patients with familial amyloid polyneuropathy. J Am Coll Cardiol.

[135] Tashima K, Ando Y, Terazaki H, Yoshimatsu S, Suhr OB, Obayashi K, Yamashita T, Ando E, Uchino M, Ando M (1999). Outcome of liver transplantation for transthyretin amyloidosis: follow-up of Japanese familial amyloidotic polyneuropathy patients. J Neurol Sci.

[136] Uehara T, Kakuda K, Sumi-Akamaru H, Yamauchi A, Mochizuki H, Naka T (2016). An autopsy case of leptomeningeal amyloidosis associated with transthyretin Gly47Arg mutation. Rinsho shinkeigaku Clinical neurology.

[137] Ueno S, Fujimura H, Yorifuji S, Nakamura Y, Takahashi M, Tarui S, Yanagihara T (1992). Familial amyloid polyneuropathy associated with the transthyretin Cys114 gene in a Japanese kindred. Brain.

[138] Umemura T, Sobue G, Morishita S, Tanaka F, Doyu M, Sakakibara T (1995). Familial amyloidotic polyneuropathy (FAP type I) with late-onset; siblings showing heterogeneity in age of onset. Rinsho shinkeigaku Clin Neurol.

[139] Vernino S, Claassen D (2017). Polypharmacy: droxidopa to treat neurogenic orthostatic hypotension in a patient with Parkinson disease and type 2 diabetes mellitus. Clin Auton Res.

[140] Wieling W, van Lieshout JJ, van Leeuwen AM (1993). Physical manoeuvres that reduce postural hypotension in autonomic failure. Clin Auton Res.

[141] Wright RA, Kaufmann HC, Perera R, Opfer-Gehrking TL, McElligott MA, Sheng KN, Low PA (1998). A double-blind, dose-response study of midodrine in neurogenic orthostatic hypotension. Neurology.

[142] Yamamoto T, Matsunaga K, Ohnishi A, Nakazato M, Murai Y (1996). A late onset familial amyloidotic polyneuropathy (FAP) with a novel variant transthyretin characterized by a basic-for-acidic amino acid substitution (Glu61– > Lys). Rinsho shinkeigaku Clin Neurol.

[143] Yazawa M, Ikeda S (1992). Autonomic nerve disorders in generalized amyloidosis. Nihon rinsho Jpn J Clin Med.

[144] Yazawa M, Ikeda S, Ushiyama M, Yanagisawa N (1991). Noradrenergic nerve fibers of the rectal mucosa in autonomic disorders: comparison of histochemical study with clinical severity and changes in plasma noradrenaline induced by standing. J Neurol Sci.

[145] Yonehara T, Ando Y, Kimura K, Uchino M, Ando M (1994). Detection of reverse flow by duplex ultrasonography in orthostatic hypotension. Stroke.

